# Phenotypic and Functional Diversity of Mast Cells

**DOI:** 10.3390/ijms21113835

**Published:** 2020-05-28

**Authors:** Satoshi Tanaka

**Affiliations:** Department of Pharmacology, Division of Pathological Sciences, Kyoto Pharmaceutical University, Misasagi Nakauchi-cho 5, Yamashina-ku, Kyoto 607-8414, Japan; tanaka-s@mb.kyoto-phu.ac.jp; Tel.: +81-75-595-4667

Mast cells, which originate from hematopoietic stem cells, are distributed in nearly all vascularized tissues. As no leukocytes that are categorized as mast cells could be found in the circulation, it is considered that the terminal differentiation of mast cells occurs under the strong influence of their microenvironment [1,2,3]. Recent studies shed light on the origin and heterogeneity of tissue human and murine mast cells [4,5,6]. The microenvironment might regulate the expression profiles of both the receptors and mediators of tissue mast cells. The sensor molecules, including the cell surface receptors expressed in tissue mast cells, determine to which types of environmental changes they should respond, while the capacity of the mediators’ release determine how they should act on these changes (Figure 1). Accumulating evidence indicates that mast cells could exert a wide variety of physiological and pathological effects in the context of spatiotemporal immune responses. Recent progress in the field of mast cell research has provided us with many powerful tools, such as a variety of gene-targeted mice lacking tissue mast cells, primary mast cell cultures, and various “omics” approaches to clarify this complexity [7], thereby enabling a comprehensive update of our knowledge about mast cell functions. The studies found in this Special Issue “Activation and Modulation of Mast Cells” are involved in this new tide of research.

IgE-mediated activation of mast cells has been intensively studied because mast cells play an essential role in IgE-mediated immediate allergic responses. Mast cells were also found to undergo degranulation in an IgE-independent manner, although it remains largely unknown how mast cells are activated by various secretagogues, such as compound 48/80 and several bioactive peptides including neuropeptides and antibacterial peptides because no suitable culture models have been developed for the in-depth investigation of IgE-independent degranulation. Recently, Staphylococcus δ-toxin was found to induce degranulation of murine mast cells, implying the important role of degranulation induced by the bacterial peptide toxin in atopic dermatitis [8]. Tatemoto et al. first proposed that a Mas-related G protein-coupled receptor subtype, MRGPRX2, should be involved in secretagogue-induced degranulation of mast cells [9]. McNeil et al. recently demonstrated that one of the murine MRGPRX2 orthologues, MrgprB2, should be responsible for IgE-independent degranulation of mast cells and various pseudo allergic responses in mice [10]. In this issue, Chompunud Na Ayudhya et al. demonstrated the functional roles of some key amino acid residues of MRGPRX2 using molecular biochemical approaches [11]. Apart from the Mrgpr family, Yoshida et al. also investigated IgE-independent degranulation of murine mast cells. They revealed that prostaglandin E_2_ and ATP could synergistically induce degranulation by acting on EP3 and P_2_X_4_ receptors, respectively [12]. Arriaga-Gomez et al. demonstrated that methylisothiazolinone could induce persistent tactile sensitivity and mast cell accumulation in female genital skin tissues [13]. Although the target molecules of methylisothiazolinone remain to be identified, it is likely that cutaneous mast cells could be directly activated by several contact allergens. Indeed, Dudeck et al. demonstrated the activation of cutaneous mast cells in the presence of several conventional contact allergens [14]. IL-33 and thymic stromal lymphopoietin were found to be potential modulators of mast cell functions [15]. Ishimaru et al. unexpectedly found that a synthetic REV-ERB agonist, SR9009, could suppress the activation of murine mast cells induced by the IgE/antigen complex or IL-33 and these effects were independent of disturbance of the circadian clock [16]. Lyons and Pullen reviewed the recent findings of IgE-independent activation of mast cells, with a focus on the context-dependent actions of TGF-β and IL-10 [17].

Diversity of phenotype and function of tissue mast cells needs more attention for a better understanding of their function. Although the concept that tissue mast cells can be categorized into two subtypes—connective tissue type and mucosal type—is commonly recognized, more detailed characterization in the context of spatiotemporal localization should be useful for elucidation of the roles of tissue mast cells. In this issue, Kakinoki et al. characterized the phenotypic changes of murine mast cells induced by IL-9, which might be essential for the development of the intestinal mast cell population [18]. Gion et al. characterized a unique population of mast cells, which may uptake IgE molecules in human eosinophilic chronic rhinosinusitis [19]. Mast cells are well known for their potential to produce a wide variety of mediators, such as biogenic amines, lipid mediators, cytokines/chemokines, and growth factors [20]. Among them, the physiological and pathological roles of mast cell proteases have emerged in recent studies using various gene-targeted mouse models [21]. Accumulating evidence suggests that the expression profiles of mast cell proteases should reflect the heterogeneity of tissue mast cells [22,23]. In this issue, Fu et al. demonstrated the specific in vitro cleavages of a series of cytokines and chemokines by mast cell proteases including human tryptase, raising the possibility that mast cell proteases modulate the direction of immune responses [24]. Ohneda et al. clarified the functional roles of GATA1 and GATA2 in transcriptional regulation of the Tpsb2 gene that encodes mouse mast cell protease 6 in murine mast cells [25].

It will be of great help for novel therapeutic approaches of inflammatory diseases to identify endogenous target molecules and synthetic compounds that could modulate the activity of tissue mast cells. Kataoka et al. summarized the modulatory roles and therapeutic potential of killer immunoglobulin-like receptor 2DL4 (CD158d) in human mast cells [26]. Uchida et al. identified a natural compound from a citrus fruit, Jabara, which could suppress degranulation and IL-6 production of murine mast cells upon IgE-mediated antigen stimulation [27].

The contents of this Special Issue reflect the diverse aspects of mast cell research. I hope that these studies will stimulate researchers and encourage further exploration in the field of mast cell research.

## Figures and Tables

**Figure 1:**
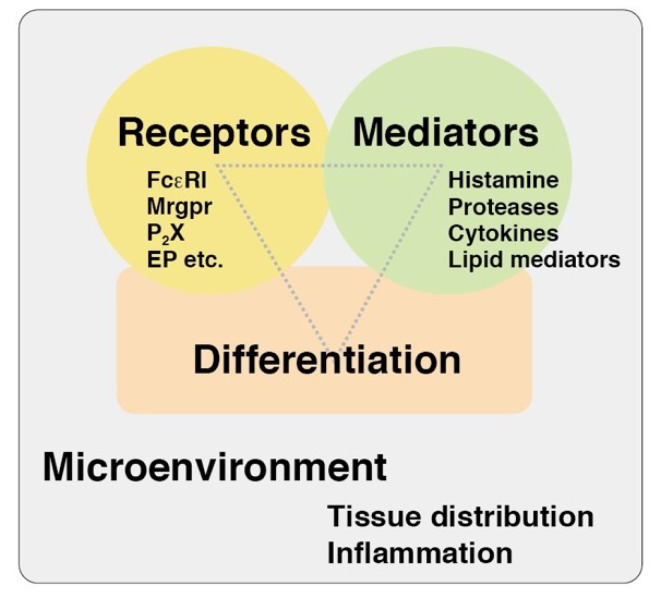
A viewpoint of mast cell research. Three viewpoints, such as differentiation states, expression patterns of the receptors, and capacities of the mediator release, might be useful for the comprehensive understanding of functions of tissue mast cells. Mrgpr, Mas-related G protein-coupled receptor; P_2_X, purine ionotropic receptor; EP, prostaglandin E receptor.
